# Cardiac Magnetic Resonance and Computed Tomography in Hypertrophic Cardiomyopathy: an Update

**DOI:** 10.5935/abc.20160081

**Published:** 2016-08

**Authors:** Diogo Costa Leandro de Oliveira, Fernanda Boldrini Assunção, Alair Agusto Sarmet Moreira Damas dos Santos, Marcelo Souto Nacif

**Affiliations:** 1 Universidade Federal Fluminense, Niterói, Rio de Janeiro, RJ - Brazil; 2 Complexo Hospitalar de Niterói, Niterói, Rio de Janeiro, RJ - Brazil

**Keywords:** Cardiomiopatia Hipertrófica, Espectroscopia de Ressonância Magnética / uso diagnóstico, Tomografia Computadorizada por Raios X / métodos, Tomografia Computadorizada por Raios X / tendências

## Abstract

Hypertrophic cardiomyopathy (HCM) is the most common genetic cardiovascular disease and represents the main cause of sudden death in young patients. Cardiac magnetic resonance (CMR) and cardiac computed tomography (CCT) are noninvasive imaging methods with high sensitivity and specificity, useful for the establishment of diagnosis and prognosis of HCM, and for the screening of patients with subclinical phenotypes. The improvement of image analysis by CMR and CCT offers the potential to promote interventions aiming at stopping the natural course of the disease. This study aims to describe the role of RCM and CCT in the diagnosis and prognosis of HCM, and how these methods can be used in the management of these patients.

## Introduction

Hypertrophic cardiomyopathy (HCM) is an autosomal dominant disorder that affects 1 in every 500 people.^1-3^ It is characterized by ventricular hypertrophy with preserved systolic function, in the absence of other conditions that may cause such changes.^4-,6^ The development of HCM is determined by mutations in genes that codify sarcomeric proteins,^4,2,7^ which cause myocyte disarray and fibrosis that are characteristic of the disease.^8-10^

Clinical manifestations of HCM range from asymptomatic patients to sudden death.^11-15^ HCM is the main cause of sudden death in adolescents, young adults and athletes.^2,3,16^ Clinically, the main risk factors for sudden death are nonsustained ventricular tachycardia, syncope, familial history of sudden death or aborted sudden death.^2,3,6^ Other risk factors include left ventricular wall thickness greater than or equal to 30 mm and left ventricular outflow tract obstruction found in echocardiography.^2-4,10^

Although the diagnosis of HCM may be established by two-dimensional echocardiography, cardiovascular magnetic resonance (CMR) has been the method of choice in the last years, due to its accuracy for determination of morphology, tissue and functional characterization, and detection of myocardial fibrosis by delayed myocardial enhancement (DME).^2,4^

Patients with HCM, in use of implantable cardioverter defibrillators (ICDs), cannot be followed-up by CMR, since the presence of ICD implant may be a contraindication for the exam. In this context, cardiac computed tomography (CCT) may be a useful alternative in the assessment and management of these patients.

This study aimed to provide an updated literature review of current concepts in the use of CMR and CCT for HCM, emphasizing the diagnostic impact of both methods.

### Interaction With Echocardiography

Echocardiography is the most available method to assess morphological and functional changes of HCM. The diagnostic criteria for HCM are left ventricular (LV) wall thickness greater than or equal to 15 mm at the end of diastole, and a septal to lateral wall thickness ratio greater than or equal to 1.3 in a non-dilated left ventricle, and in the absence of other conditions that may explain such abnormality.^17-19^

Although widely available, the method has some limitations in the evaluation of HCM, such as: patients with poor acoustic window, poor visualization of some regions - basal anterolateral wall of left ventricle, cardiac apex and right ventricle (RV).^2,8,20,21^ Both CRM and CCT are three-dimensional, multiplanar methods, with excellent spatial resolution, that have been recognized as important tools for the assessment of HCM patients.^2,22^

### Cardiac Magnetic Resonance

CMR is an excellent method for the evaluation of HCM, since it precisely determines both the localization and extension of hypertrophy and evaluates ventricular function. The method also allows the detection of mechanisms of obstruction of the LV outflow tract, as well as the establishment of the pressure gradient between the LV outflow tract and the aorta. Other advantages include detection of areas of myocardial fibrosis by DME, diagnosis of apical HCM, and the follow-up of patients undergoing septal ablation.^2,10,21,22^

CMR is more sensitive than echocardiography in the detection of HCM markers, such as myocardial crypts.^20,22^ It can contribute to the preoperative planning of myectomy and quantification of necrotic tissue by alcohol septal ablation. Due to these characteristics, CMR has been considered a method to be routinely used for the establishment of diagnosis and prognosis of HCM.^20^

#### Genetic variants (genotype)

HCM is an autosomal dominant genetic disorder that determines mutations in genes that codify sarcomeric proteins.^6,8^ The most frequent mutations involve beta-myosin heavy chain gene (MYH7), myosin-binding protein C (MYBPC3) and troponin T (TNNT2).^6,7^ Mutation in MYH7 is associated with earlier manifestations of HCM as compared with MYBPC3^7^ gene mutation, whereas TNNT2 gene mutation is more associated with the risk of sudden death.^6^

Until recently, the presence of reverse septal curvature was the only change in CMR imaging that indicated mutations in genes that codify sarcomeric proteins. However, studies conducted in 2012 identified deep basal inferoseptal crypts as stronger positive predictors of HCM genotype, since they can be found in 81% of patients who are genotype-positive for HCM.^23,24^ Besides, the combination of both changes increases the positive predictive value (98%) for mutation.^25^

Recent studies have demonstrated that other changes are indicative of the presence of a sarcomere gene mutation, including anterior mitral valve leaflet elongation, abnormal trabeculae and smaller LV systolic cavity.^26^ The identification of these abnormalities allows the detection of individuals who are carriers of mutations, and hence more susceptible to the development of HCM. This information enables the development and implementation of strategies that can change the natural history of HCM,^7,25^ as well as the development of new imaging sequences that better characterize such changes.^25^

#### Morphological variants (phenotype)

**Figure 1:**
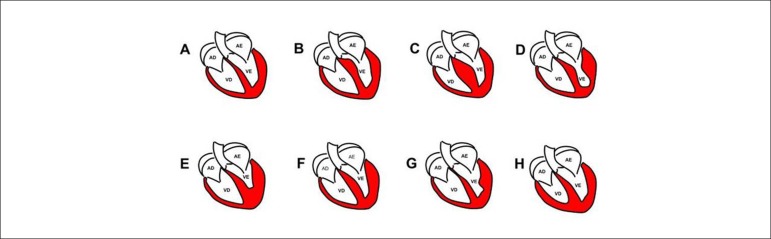
Types of hypertrophic cardiomyopathy. (A) Normal heart. (B) Sigmoid septum. (C) Reverse septal curvature. (D) Mid-ventricular obstruction without apical aneurysm. (E) Apical. (F) Symmetric. (G) Asymmetric hypertrophy in the lateral wall. (H) Hypertrophic cardiomyopathy in right ventricle.

The precise characterization of the HCM phenotype is valuable for the establishment of invasive therapies, including septal myectomy and alcohol septal ablation. The HCM phenotype allows the definition of localization and magnitude of hypertrophy,^21^ and characterization of mitral and submitral apparatus and papillary muscles.^20^ Many morphological variants of HCM have been described using CRM (Figure 1).

- **Normal variant** - related to relatives of indiviuals genetically positive for HCM, or those who should be followed up for the risk of developing any other variant throughout life. We should consider the presence of crypts 24, abnormal trabeculation, and anterior mitral valve leaflet elongation.^26^
- **Asymmetric variant with sigmoid septum** - this is the main presentation of CMH,^6,16^ characterized by a myocardium hypertrophy next to the LV outflow tract and the sigmoid septum ("S" shape). This variant may cause subaortic obstruction and mitral regurgitation.
- **Asymmetric variant with reverse septal curvature** - characterized by a septum hypertrophy as a reversed "S", more distant from the LV outflow tract. This presentation does not cause obstruction to the LV outflow. The identification of this variant by CMR is characterized by a septal/free wall thickness ratio greater than 1.3 in the short-axis plane.^4^
- **Variant with mid-ventricular obstruction with or without a left ventricular apical diverticulum** - characterized by a mid-ventricular hypertrophy that causes a local narrowing and, in severe cases, apical dilatation. In approximately 10% of patients, there may be apical aneurysm formation.^4^ Apical aneurism is better diagnosed by CRM than echocardiography which, in turn, can fail to detect this change in 10% of cases.^16^
- **Apical variant** - characterized by obliteration of LV cavity at the apex together with an apical wall thickness greater than 15 mm or a ratio between apical and basal LV wall thicknesses greater than or equal to 1.3-1.5 cm^4^. This presentation is considered to have a better prognosis than the other variants, although it has been more associated with ischemia and apical myocardial infarction^16^ (Figure 2).
  **Figure 2:**
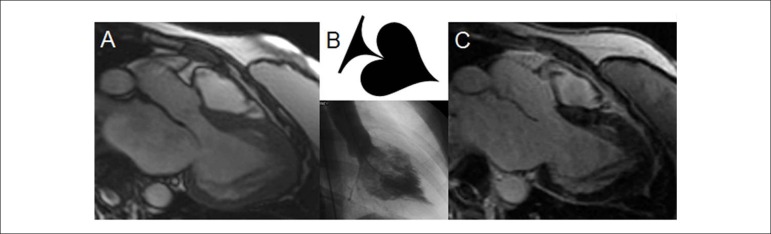
Apical hypertrophic cardiomyopathy. Parietal hypertrophy, predominantly apical, with the ‘ace of spades’ sign, described in ventriculography studies. Absence of myocardial fibrosis in the delayed enhancement technique. (A) Cine-magnetic resonance. (B) Ace- of-spades sign by ventriculography and (C) Delayed myocardial enhancement.
- **Symmetric (concentric) variant** - characterized by symmetric hypertrophy of LV wall with reduction of LV cavity. This variant may also be present in other disorders, including amyloidosis, sarcoidosis and Fabry's disease^4,10^ (Figure 3).
  **Figure 3:**
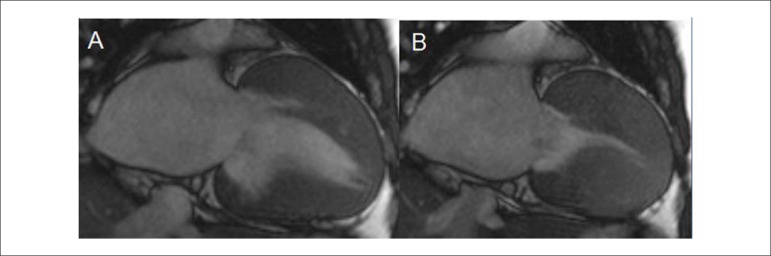
Symmetric hypertrophic cardiomyopathy. Diffuse parietal hypertrophy of left ventricle. (A) Diastole, cine-magnetic resonance and (B) Systole, cine‑magnetic resonance
- **Focal variant** - characterized by hypertrophy located in the myocardium. CMR help distinguishing focal HCM from other cardiac masses, by identifying evidence of myocardial contractility in the former case.^4^
- **HCM in RV** - occurs in 18% of HCM patients, generally involving the mid-and apical portion of the RV. This may cause right ventricular outflow obstruction in severe situations^4^. Increased maximum thickness of the right ventricular wall (> 8 mm) has been shown by CMR in approximately 20% of HCM patients.^10^ Areas with increased wall thickness are commonly observed in the insertion of the right ventricular wall into anterior and posterior septum, although the entire RV may be involved.

#### Assessment of ventricular mass

LV hypertrophy is currently one of the clinical requirements for HCM diagnosis, and is associated with poorer prognosis.^4^ However, comparative studies of genotypes and phenotypes involved in HCM have reported that varied LV wall thickness may be found in patients with HCM.^27^

A reliable, accurate, quantitative analysis of LV wall thickness is fundamental, since a measurement greater than 30 mm increases the risk of sudden death.^28-30^ Therefore, this may be a crucial information for the implant of ICD to prevent sudden death in some patients. Measurements of LV wall thickness should be performed in the short-axis plane, at the end of diastole.^10^ LV mass, quantified by CMR (indexed for body surface area), greater than 2 standard deviations above the normal range is considered a sensitive predictor of favorable clinical outcomes in HCM. The normal range for LV mass index is 62.5 ± 9.0 g/m^2^ for men and 54.6 ± 12.0 g/m^2^ for women.^10^ However, the measurement of LV mass lacks specificity as an indicator of clinical outcomes, probably because in many HCM patients, hypertrophy is limited to a small number of segments of the left ventricle.^29^

**Figure 4:**
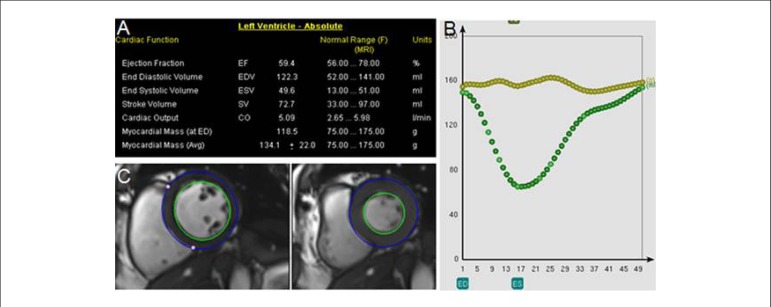
Assessment of systolic and diastolic mass and function by Simpson’s technique and time-volume. (A) Quantification of left ventricular mass, function and volume. (B) Volume-vs.-time curves and left ventricular mass with variation lower than 5%. (C) Quantification of cine-magnetic resonance during systole and diastole.

CMR is more accurate than echocardiography in diagnosing ventricular hypertrophy, its magnitude and distribution.^21^ For this reason, the method is decisive for stratifying the risk of HCM. In addition, CMR provides a better evaluation of hypertrophy distribution, especially when it is localized in the anterolateral region, and in posterior and apical septum of LV,^3,10,20^ and a more reliable quantification of the myocardial mass in case of asymmetric hypertrophy^10,15,31^ (Figure 4).

#### Quantification of myocardial fibrosis

Myocardial fibrosis can be detected by delayed enhancement CMR. This method is based on the property of gadolinium to distribute in the extracellular space between normal and fibrotic tissue, leaving the latter tissue in a slower rate.^2^

There is no standard of reference for DME in HCM, although its presence in the interventricular septum, particularly in the mid- and basal anteroseptal segment, is suggestive of HCM.^10^ DME can also occur in LV free wall and hypertrophied segments of the ventricles. These data indicate that analysis of the segments by delayed enhancement CMR is an important parameter for differential diagnosis of HCM.^4,22^

DME is found in 65% of patients and the distribution of fibrosis typically follows a multifocal, heterogeneous and mid-wall pattern.^2^ DME is not commonly found in non-hypertrophic cardiomyopathy, except in advanced stages of the disease, and can be associated with increased myocardial stiffness and reverse remodeling of the left ventricle.^4,20^ Studies have demonstrated an inverse relationship between DME and LV function.^4^

**Figure 5:**
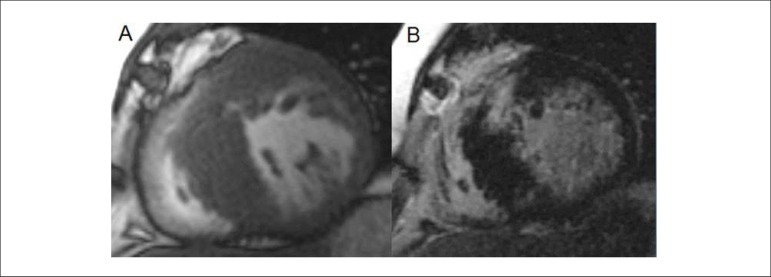
Asymmetric hypertrophic cardiomyopathy. Asymmetric parietal hypertrophy with septal predominance. Presence of important myocardial fibrosis in the mid anteroseptal segment (white) in the delayed enhancement technique. (A) Cine-magnetic resonance and (B) Delayed myocardial enhancement.

The presence of DME predicts a worse prognosis for HCM patients due to higher risk of sudden death, systolic dysfunction and nonsustained ventricular tachycardia.^32-34^ Areas of DME may represent the substrate for malignant ventricular tachyarrhythmias^10,35^ (Figure 5). Delayed enhancement CMR is a supporting tool in the decision-making process for primary prevention ICDs in patients in whom high-risk for sudden death remains uncertain after assessment of conventional risk factors.^35^

**Figure 6:**
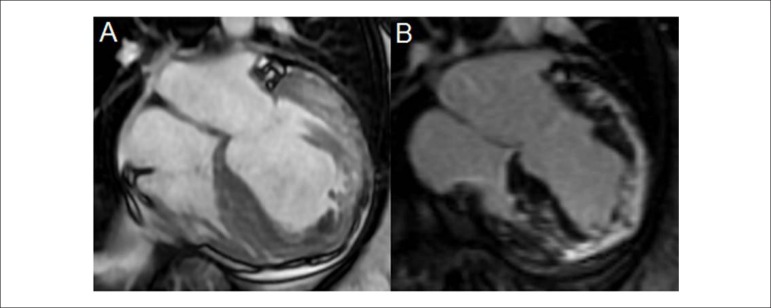
End-stage hypertrophic cardiomyopathy. Diffuse parietal hypertrophy, and hypertrophy in both ventricules, associated with dilation and important dysfunction. Presence of well-defined myocardial fibrosis (white) in delayed enhancement technique. (A) Cine-magnetic resonance and (B) Delayed myocardial enhancement.

DME technique consists in the intravenous administration of approximately 0.2mmo/kg of gadolinium-based contrast at 1-2 mL/s. Acquisition of images may be started 10 minutes after contrast infusion, at multiple inversion times (TI Scout), which allows the use of a proper TI to null the myocardial signal (typically between 200 and 300 s). DME images (1 cm-sections) are acquired in the short-axis plane, from the base to the apex of the heart, using a T1-weighted inversion recovery gradient-echo sequence. Images in two- and four-chamber plane were also acquired, for better visualization of apical disease^10,34^ (Figure 6).

Recent studies^36,37^ have pointed out that the presence of either extensive late enhancement or late enhancement in the interventricular septum and right ventricular anterior and posterior insertions may be a biomarker of sudden death. Patients in this situation should be classified as high risk. We recommend that quantification of myocardial fibrosis should be performed with visual assessment.

#### Analysis of diastolic dysfunction

**Figure 7:**
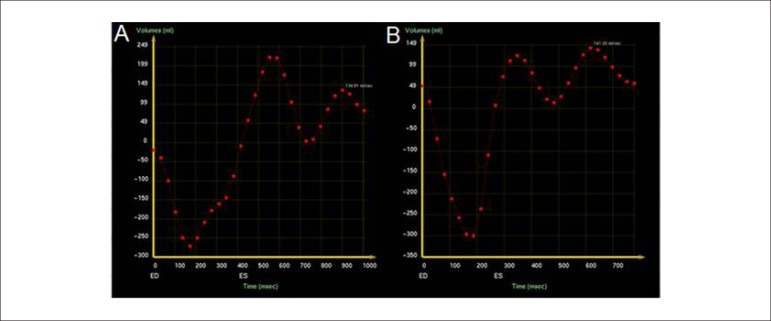
Assessment of (derivative) volume curves to quantify diastolic dysfunction (A) Preserved relationship between rapid filling phase and atrial contraction curves. Rapid filling time was 580 ms and atrial contraction occurred in 900 ms. (B) Type I diastolic dysfunction with atrial contraction amplitude greater than rapid filling. In this case, rapid filling time was 375 ms and atrial contraction occurred in 615 ms.

Diastolic dysfunction in HCM results from abnormal dissociation of actin and myosin filaments during diastole, mainly in its early active phase.^30,38^ It can be measured by time-volume curve obtained from cine-magnetic resonance imaging^4^ (Figure 7).

Diastolic function can also be evaluated by in-plane phase-contrast CMR imaging, with direct measurement of diastolic relaxation of cardiac muscle. This, in turn, may be obtained by the assessment of LV strain and recovery rates during diastole,^4,30,38^ yielding the values of fast relaxation (E') and slow relaxation (A'). In hypertrophied segments, the early diastolic filling velocity is reduced, and there is a decrease in the rate of LV relaxation.^4,38^

Mitral valve flow velocity and pulmonary artery flow velocity can also be estimated by through-plane phase contrast, allowing the calculation of pressure gradient and flow velocity.^4^ Analysis of transmitral flow enables the estimation of early ventricular filling velocity (E-wave) and peak flow velocity at atrial contraction (A-wave). Mild diastolic dysfunction is characterized by a reduction of E/A ratio. As the dysfunction progresses, there is a pseudonormalization and subsequent increase in the E/A ratio, which represents the restrictive physiology of diastolic dysfunction.^10,38^

#### Use of myocardial tagging (MT)

**Figure 8:**
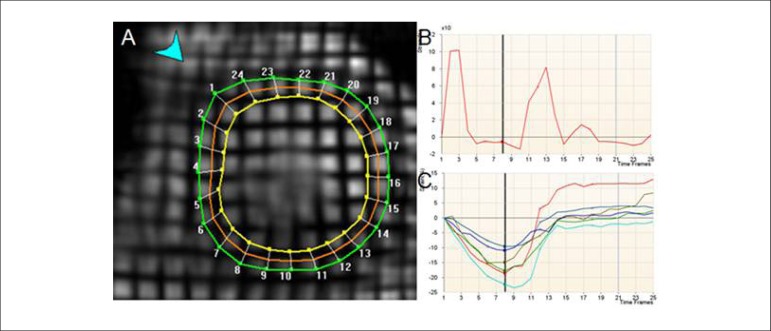
Myocardial tagging for quantification of systolic and diastolic function in left ventricle. (A) Subendocardial, mesocardial and epicardial trace. (B) Global radial strain. (C) Segmental circumferential strain.

Myocardial tagging (MT) is an excellent non-invasive tool for quantifying regional diastolic and systolic myocardial function.^39^ Quantification of global myocardial dysfunction is not sensitive to detect small reductions in regional ventricular performance, which may occur even in normal LV ejection.^40,41^ In this context, MT has emerged as a technique that allows the definition of subclinical myocardial dysfunction (Figure 8).

MT evaluates myocardial deformation during the cardiac cycle. The most used method is a coordinate system,^42^ which takes into account three axes of myocardial contraction: radial, circumferential, and longitudinal.^39,42^

- **Radial strain** - describes myocardial stiffness, oriented toward the central long axis of the left ventricle.
- **Circumferential strain** - describes circumferential shortening of myocardium, in the short-axis plane, tangential to the epicardial wall.
- **Longitudinal strain** - represents LV shortening from the base to the apex, in the long-axis plane.
- **Myocardial rotation** - represents the analysis of mid-myocardial deformation, in degrees, in the short-axis plane during systole or diastole. In a normal cardiac cycle, myocardial rotation is generated by a clockwise rotation of the base and a counterclockwise rotation of the apex during systole. During diastole, the opposite rotation to the normal position promotes the release of potential energy stored, and a suction force in the left ventricle during isovolumic relaxation. A change in the rotation pattern may be used to detect subtle systolic and diastolic dysfunctions in many cardiac diseases.^39^ Myocardial twist (in degrees) can be calculated as the diference between apical and basal rotation during all cardiac cycle. We can calculate torsion (in degrees per cm) as a type of twist normalization by ventricular longitudinal length. Also, the peak of systolic or diastolic rotation, twist and torsion, can be also calculated, but they are only used in scientific studies.

#### Assessment of the LV outflow track

Obstruction of LV outflow tract (induced or at rest) is found in approximately 70% of patients with HCM.^4^ During systole, LV outflow is hampered by basal septal hypertrophy and displacement of the papillary muscles and mitral leaflets.^16^

Obstruction of LV outflow with a maximum gradient at rest greater than 30 mmHg is a strong predictor of sudden death. In this case, interventions to reduce such gradient, including septal miectomy and alcohol septal ablation by catheterization, may be justified.^10,43^

Two-D transthoracic echocardiography is currently the method of choice for anatomic assessment and flow measurement in case of LV outflow obstruction.^8,10,22^ However, CMR enables a more accurate evaluation of the mitral valve structure, including changes in the papillary muscle anatomy.

Although the quantification of the LV outflow obstruction gradient may be performed by CMR, it still represents a challenge, since it requires an accurate image plane alignment to prevent the loss of high-velocity areas. Besides, the presence of turbulent outflow causes signal loss and phase error.^10^ New sequences in ultrashort echo time CMR imaging have been currently used and may make the evaluation of LV outflow obstruction easier and more reliable.^10^

#### Assessment of myocardial ischemia

Myocardial ischemia caused by microvascular disease is an etiopathogenic hypothesis for the development of HCM.^22^ For this reason, the tests for myocardial ischemia represent a valuable tool for event risk stratification.

Myocardial perfusion techniques using gadolinium administration via an infusion pump during pharmacologic stress and at rest are excellent methods for the diagnosis of subendocardial ischemia.^43^ The early detection of ischemia before cardiac remodeling may represent a promising therapeutic target that can change the natural history of HCM.^10^

#### Contraindications and limitations

CMR imaging in HCM patients depends on the technical quality of image acquisition and requires interpretation of the images by experienced physicians.^20,35^ The method has some relative and absolute contraindications, which have been reassessed every year. This includes the performance of CMR in patients with pacemaker, ICD, brain clips, cochlear implants, and metallic fragments in the eyes.^2^ Today, the test can be performed in some pacemakers and patients.^44^

Limitations of CRM include nephrogenic systemic fibrosis (which causes systemic tissue fibrosis and is associated with the use of gadolinium in patients with stage 4 and 5 chronic renal failure) and patients with hepatorenal syndrome.^45^ In addition, it is worth mentioning that, despite the increase in accessibility to the CRM, the test is available in a small number of centers.

### Cardiac Computed Tomography

CCT provides a clear delineation of the myocardium and accurate measurement of cardiac wall thickness, ventricular volumes, and LV mass ejection fraction, which are well correlated with the CMR findings. Additionally, CCT allows the assessment of coronary arteries and cardiac valves. The European Guidelines on HCM recommends that CCT should be considered in patients with poor acoustic window for echocardiography or contraindications for CMR (class IIa).^20^

CCT has a wide range of clinical applications due to anatomic and functional properties. However, they are indicated only in case of diagnostic doubt, poor acoustic window to perform echocardiography or contraindications to perform CMR. Therefore, CCT is rarely used as the first method of choice for patients with HCM.^43^ Table 1 describes the main clinical applications of CCT in HCM.^43^

**Table 1:** Clinical applicability of computed tomography in the assessment of hypertrophic cardiomyopathy

	**Parameters**	**Clinical applicability**
Ventricles	Diameters / volumes	Indicated in case of diagnostic doubt
	Wall thickness	
	Global systolic function	
	Regional systolic function	
	Diastolic function	Not indicated
Atriums	Volumes	Indicated in case of diagnostic doubt
	Function	
Valves	Systolic anterior motion	It may be indicated
	Mitral regurgitation	Not indicated. It may be used only for static evaluations, such as valve planimetry and morphological characterization.
Pharmacological stress	Ischemia / perfusion defects	It may be indicated especially in combination with anatomical features of coronary arteries
Coronary	Luminal reduction	Deve ser utilizada
	CAD	
Pressures / Velocities	Gradients	Not indicated
Fibrosis	Delayed myocardial enhancement	It may be used in case cardiac magnetic resonance is contraindicated

Adapted from Nagueh et al.^43^. CAD: coronary artery disease.

#### Delayed myocardial enhacement

Since the demonstration that CCT allows the visualization of fibrotic areas in patients with acute myocardial infarction similarly with CMR,^46,47^ a similar DME technique has been developed to identify fibrosis in HCM patients.^48^ Studies have shown a good correlation between DME by CCT and DME by MCR.^49^

DME technique should be performed in multidetector computed tomography scans, capable to connect cardiac acquisition to electrocardiography. This is the case of some of the 16-channel multidetector scanner and most of the 64-channel devices. Iodinated contrast (150 mL) is intravenously administered by automatic injector at 3 mL/s. Seven minutes later, the images are acquired in retrospective ECG-synchronized helical mode, during a 10-second breath-holding, and rebuilt during the same diastolic cycle.

#### Contraindications and limitations

In comparison with CMR, CCT has inferior temporal resolution and soft tissue characterization.^10^ Radiation exposure from a 64-channel computed tomography scanner (mean of 6.7 ± 2.07 mSv)^50^ is a drawback of the method as compared with CMR. Nevertheless, the development of new techniques and CCT scanners involving lower radiation doses may provide safer conditions for myocardial fibrosis investigation.^49^

### Perspectives

#### T1 mapping by CMR

Conventional techniques to assess DME that evaluates the presence of focal myocardial fibrosis may underestimate the distribution and extension of fibrosis. The technique of T1 mapping is more sensitive to detect fibrotic areas, since it amplifies regional variations in gadolinium distribution, making it easier to detect diffuse interstitial myocardial fibrosis.^8,10,22^

Recent studies have demonstrated that HCM genotype- and phenotype- positive patients, and genotype-positive / phenotype-negative patients have increased levels of biomarkers of collagen deposition. This indicates that increased levels of profibrotic markers are also found in patients without ventricular hypertrophy, suggesting that hypertrophy is preceded by fibrosis process.^7,51^ In this case, T1 mapping may be used at the initial phase of HCM for the early detection of diffuse myocardial fibrosis in these patients.^51^

Today, the modified Look-Locker inversion recovery (MOLLI) has been the most studied sequence for the assessment of T1 mapping. MOLLI uses electrocardiogram-gated image acquisition at end-diastole and merges 11 images in a 17-heartbeat breathhold. Many protocols have been published and may be used in the assessment of interstitial myocardial fibrosis by CMR.^52^

Recently, Nacif et al.^52^ have developed a method to quantify interstitial fibrosis by CCT that may be useful in HCM.

#### Spectroscopy

Spectroscopy by 31 P-CMR may be used to assess the energy status in the myocardial tissue. Patients with HCM have decreased myocardial energy status that directly correlates with extension of hypertrophy and severity of diastolic function.^53^ Limitations of spectroscopy include prolonged scanning time, low spatial resolution and requirement of dedicated surface coils.^10^

#### Aortic stiffness

The assessment of aortic stiffness may be an important parameter for risk stratification in HCM.^42^ Studies have demonstrated that HCM patients have increased aortic stiffness, especially those with myocardial fibrosis detected by DME technique.^10^

## Conclusion

HCM is the most common genetic cardiovascular disease with potential for high mortality, since it is the most common cause of sudden death in young patients. The development and improvement of imaging analysis by CMR and CCT has enabled the early establishment of diagnosis and prognosis of HCM. In the future, interventions aimed at stopping the natural history of the disease can be made. Both CMR and CCT are validated as methods with high sensitivity and specificity, with few contraindications and minimal risks of adverse effects, and should be used in the management of patients with HCM.
